# Phase Dependence of Surface Charge Measurement on Epoxy Insulator in C_4_F_7_N/CO_2_ under AC Voltage

**DOI:** 10.3390/polym16182585

**Published:** 2024-09-13

**Authors:** Shuangying Li, Yu Gao, Di Lu, Pinhao Huang, Boxue Du

**Affiliations:** School of Electrical and Information Engineering, Tianjin University, Tianjin 300072, China

**Keywords:** GIS, epoxy insulator, surface charge, C_4_F_7_N/CO_2_, truncated phase

## Abstract

The application of binary gas mixtures consisting of heptafluorobutyronitrile (C_4_F_7_N) and carbon dioxide (CO_2_) in AC GIS is currently attracting much attention. Therefore, the evaluation of the gas–solid interface charge distribution characteristics of epoxy resin is indispensable. Additionally, the phase-dependency of the charging behavior remains not fully understood. We simulated coaxial electrode structure in GIS and investigated the surface charge distribution on a down-scaled epoxy insulator. The influence of the truncated phase angle and duration of AC voltage on charge behavior were analyzed, and the charge transport mechanism under AC voltage was theoretically analyzed. The results showed that there was a noticeable negative charge speckle with the presence of the metal particle and the accumulated negative charge on the insulator surface far exceeded that of the positive charge. The maximum surface charge density and the amount of surface charge accumulated first increased and then decreased over time. It was found that the phase angle has a negligible influence on the surface charge distribution at the cut-off moment.

## 1. Introduction

Gas-insulated switchgear (GIS) is the critical infrastructure in power transformation systems, with its operational reliability directly impacting the safety of power supply [1,2]. As essential components, insulators play the role of electrical isolation and mechanical support [3]. Given the recurring occurrence of abnormal discharge faults along insulator surfaces in GIS operations, extensive investigations have been conducted, among which insulator flashover failures accounted for the vast majority [4,5]. It is agreed that under the influence of high-voltage (HV) electric field, free charges will be accumulated at the insulator–gas interface to form surface charge [6,7,8]. On the one hand, these charges will distort the surface electric field and induce partial discharge; on the other hand, they also act as seed charge for the development of surface discharge, facilitating the onset of the flashover [9].

Currently, research on the mechanism of charge accumulation under DC voltage and the influence of charge accumulation on surface flashover has been widely carried out. Under long-term DC electric field forces, the surface treatment of insulators, the optimization of insulator structure to improve electric field distribution, and the prevention of contaminant deposition can be employed to regulate surface charge and enhance insulation performance [10,11,12]. In comparison, there is relatively little research on the charge accumulation characteristics under AC voltage [13]. However, in recent years, there have been numerous unexplained sudden flashovers of AC GIS equipment in engineering, and its discharge inducement and mechanism are still uncertain [14]. P. Morshuis et al. studied the accumulated charge behavior on the surface of cylindrical insulators in SF_6_ gas under AC voltage. It showed that the charge on the insulator surface is mainly negative, but the charge accumulated is less than that under DC or impulse voltage [15]. S. Tenbohlen found that the charge distribution on the insulator surface under AC voltage was correlated with the voltage phase, and the charge near the electrode exhibited the same polarity as the voltage during the charging period [16]. However, research into the characteristics of surface charge accumulation remains incomplete, particularly under long-term operation with the presence of factors such as highly non-uniform electrical field and so on. In general, there is no consistent conclusion on the charging mechanism under AC voltage, and no effective suppression method can be proposed.

C_4_F_7_N is a newly popular insulation gas with a dielectric strength roughly double that of SF_6_ and a significantly low GWP of 2090 [17]. However, its high liquefaction temperature (−4.7 °C) necessitates blending with buffer gasses like CO_2_ to meet the minimum operating temperature requirement of the equipment [18]. The mixed gas exhibits favorable performance characteristics and is environmentally friendly, presenting itself as a viable candidate to supplant SF_6_ across various types of equipment. J. Wang et al. investigated the accumulated charge and flashover of the insulator surface in C_4_F_7_N/CO_2_ mixtures by applying DC superimposed impulse voltage. They observed that with a metal particle attached to the insulator, the surface potential would be significantly distorted, leading to flashover occurring along the attachment position [19]. D. Li et al. found that the accumulated surface charge presented a three-layer concentric circle shape in C_4_F_7_N/CO_2_ mixed gas under AC voltage by employing a needle-plate electrode, and the polarity of the inner circle was related to the cut-off phase [20]. Scholars have conducted relatively comprehensive investigations on the surface flashover characteristics of C_4_F_7_N/CO_2_ mixtures under various voltage shapes [21,22]. As for how the truncation phase of AC voltage affects the surface charge distribution of insulators, there is no corresponding research conclusion on this issue. Previously, when most scholars studied the surface charge accumulation characteristics of solid insulation under AC voltage, they only measured the final distribution of surface charge after the voltage was removed, and did not consider the effect of the phase of AC voltage on charge accumulation. Or because the phase of AC voltage cannot be accurately controlled when it is powered off, there are few relevant research conclusions.

This paper investigated the distribution of surface charge under 20 kV AC voltage in C_4_F_7_N/CO_2_ mixtures on the down-scaled disk epoxy insulator sample, and theoretically analyzed by simulation method. The charge accumulation effect was more obvious with the presence of a metal particle [23]. The results revealed the maximum surface charge density and the amount of surface charge accumulated first increased and then decreased over time. The phase angle was found to have a negligible influence on the surface charge distribution characteristics.

## 2. Experimental Setup and Methods

### 2.1. Insulator Sample and Electrode Structure

**Figure 1:**
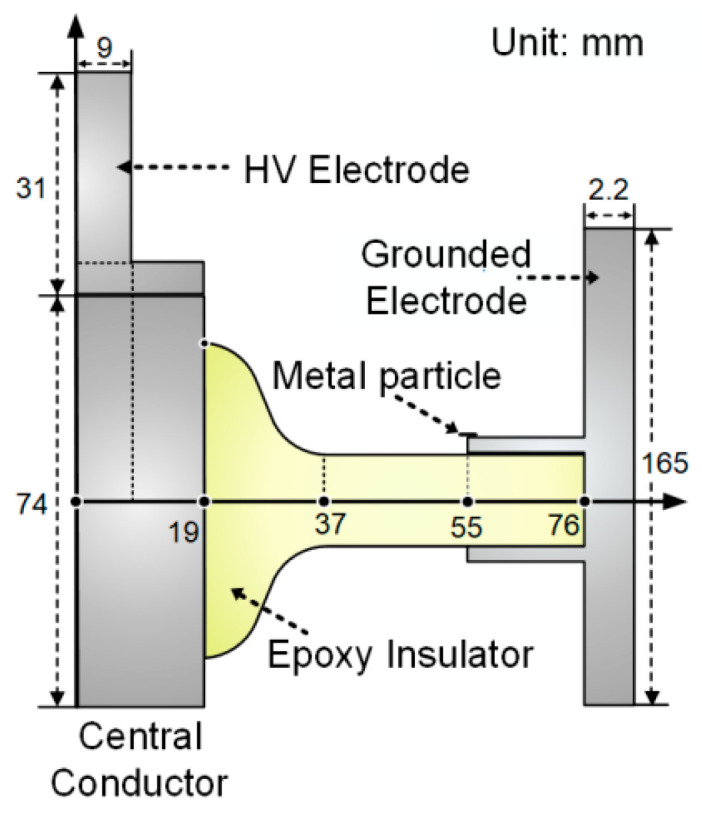
Schematic diagram of the test insulator and coaxial electrode.

The arrangement of the insulator sample and electrodes is depicted in Figure 1. To facilitate the comparative analysis of subsequent experimental results, the test insulator was designed following the shape of the disk-shaped insulator suitable for 126 kV GIS, scaled down to 44% of the original size after equivalent field calculation [24]. The sample was manufactured by Taikai Group Co., Ltd., Tai’an, China, and was obtained by doping epoxy resin and Al_2_O_3_ at a mass ratio of 3:1. The central conductor and grounded electrode were arranged in a coaxial arrangement to simulate the electric field distribution on the insulator surface in GIS. A linear aluminum metal particle with a diameter of 0.5 mm and a length of 10 mm was affixed to the grounded electrode and was extended by 5 mm to suspend above the insulator. The boundary between the planar region (close to the grounded electrode) and the non-planar region (close to the HV electrode) was set at a distance of 37 mm from the center of the central conductor.

### 2.2. Experimental Circuit

**Figure 2:**
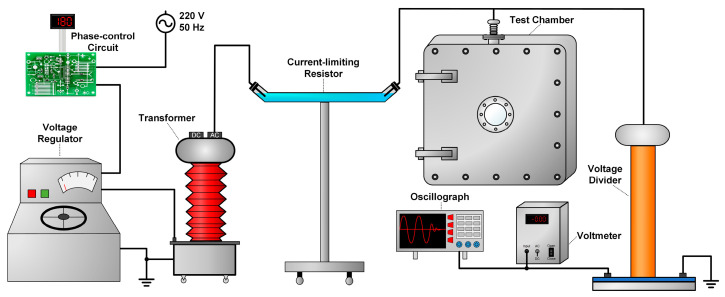
Schematic diagram of the HVAC charging circuit.

The HVAC charging system shown in Figure 2 consists primarily of a phase-control circuit, a voltage regulator (250 V 10 kVA), a transformer (1:1000), a current-limiting resistor (6 MΩ), a chamber with an HV electrode, a voltage divider (1000:1), and a voltmeter and an oscilloscope. To realize the controllable switching of power frequency voltages, the phase-control circuit featuring a single-chip microcomputer (STC8H1K08, Hongjing Technology Co., Ltd. Shenzhen, China) was designed for this experiment. The circuit was controlled to cut-off the applied voltage at a specified phase angle (0° to 359°). The carried relay’s (SHV05-1A85-78D3K, Standex-Meder, Germany) operation time was less than 0.1 ms with an accuracy error of less than 0.5%. The voltage regulator and transformer were utilized to adjust the voltage, while the current-limiting resistor prevented equipment damage from excessive discharge current. The real-time monitoring of the charging voltage is facilitated by the voltage divider and oscilloscope.

### 2.3. Experimental Platform and Method

**Figure 3:**
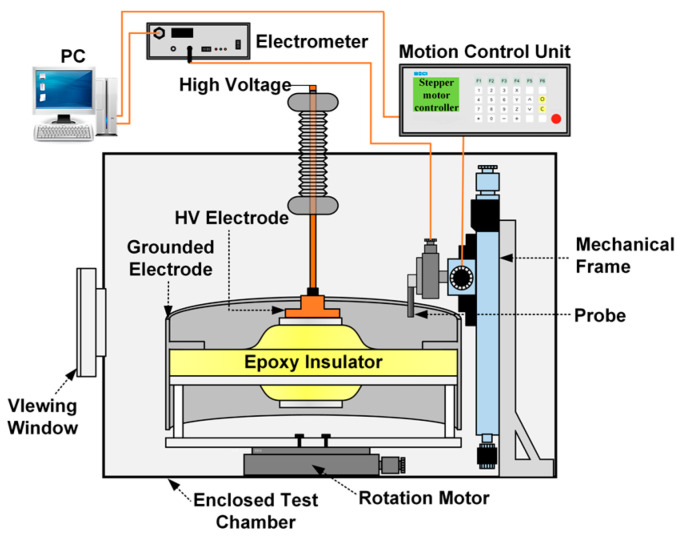
Structure of charging and measurement system.

Figure 3 shows the charging and measurement device. The enclosed test chamber was utilized to regulate the internal gas pressure to 0.1 MPa, and was filled with a mixture of 10% C_4_F_7_N and 90% CO_2_. The insulator was positioned on a rotation motor and charged via the HV electrode. The insulator could rotate 360° under the control of the rotating motor. The Kelvin probe (3455ET, Trek, USA) was positioned on a mechanical frame and driven by a displacement motor for three-dimensional motion. By coordinating the rotation of the insulator and the radial movement of the probe along the insulator surface, the 648 prepositioned surface potential values can be obtained.

The AC voltage of 20 kV was applied to the central conductor. Four voltage durations of 2, 5, 30, and 60 min were chosen to investigate the change in accumulated charge. The surface potential of the insulator was measured after the voltage was cut-off at phase angles of 0°, 90°, and 180° respectively. The measured potential was converted into charge density by the inversion algorithm and the charge density distribution was obtained to reflect the accumulation characteristics of surface charge [25].

## 3. Experimental Results

### 3.1. Influence of Phase Angle on Surface Charge Distribution

**Figure 4:**
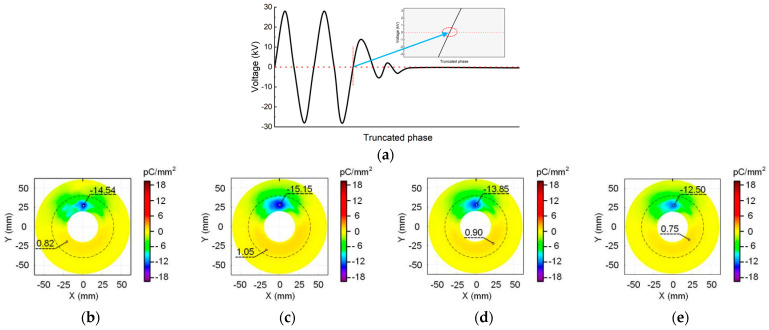
Surface charge distribution at the cut-off phase angle of 0°: (**a**) waveform diagram; (**b**) 2 min; (**c**) 5 min; (**d**) 30 min; (**e**) 60 min.

**Figure 5:**
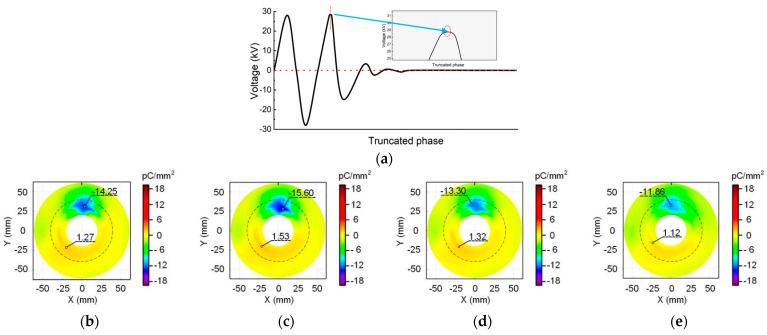
Surface charge distribution at the cut-off phase angle of 90°: (**a**) waveform diagram; (**b**) 2 min; (**c**) 5 min; (**d**) 30 min; (**e**) 60 min.

**Figure 6:**
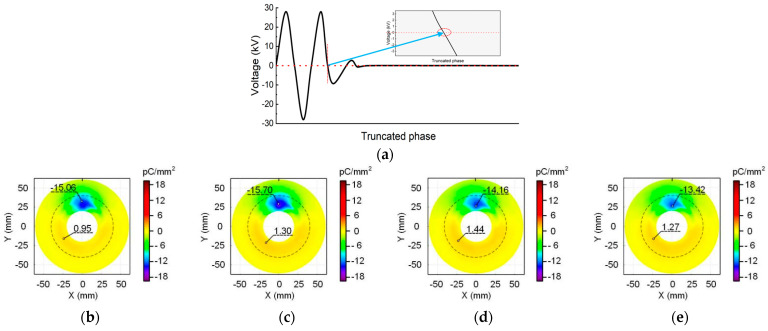
Surface charge distribution at the cut-off phase angle of 180°: (**a**) waveform diagram; (**b**) 2 min; (**c**) 5 min; (**d**) 30 min; (**e**) 60 min.

The phase-control circuit described in Section 2.2 is utilized to cut the HV sinusoidal wave at different phase angles. Three specific angles are chosen: 0° corresponds to the negative zero-crossing moment of the voltage, 90° corresponds to the peak value of the positive voltage, and 180° corresponds to the positive zero-crossing moment of the voltage. The waveform of the voltage cut-off time is captured by the oscilloscope and depicted in the schematic diagram shown in Figure 4a, Figure 5a and Figure 6a. Following voltage cessation, the transformer coil induces oscillations in voltage amplitude, gradually reducing to zero. The angle where the waveform oscillation starts is marked by a red dotted line. The positions and corresponding values of maximum positive and negative charge densities are highlighted in the charge distribution diagram. Where the metal particle appears is depicted by a black short line at the top of the figure. The boundary between the planar and non-planar regions of the insulator surface is delineated by a dashed circle.

Figure 4 shows the surface charge distribution at the cut-off phase angle of 0°. The negative charge speckle appears consistently in the non-planar region near the metal particle regardless of when the voltage is cut-off. Moreover, the position and shape of the speckle remain unchanged over time. Far away from the metal particles, a small amount of positive charge accumulates in the non-planar region. In other planar regions distant from the charge speckle, the charge density approaches zero. However, both the maximum charge density value and the size of the charge speckle area show an increasing trend within the initial 5 min of voltage application. When the voltage duration exceeds 30 min, the accumulated charge speckle on the insulator surface tends to dissipate.

The results in Figure 5 are derived from the cut-off phase angle of 90°. It can be seen that there is also a negative charge speckle towards the metal particle, and the distribution positions of the positive and negative charges are the same as that in Figure 4.

In addition, it shows that as the voltage duration increases, the maximum charge density value and charge speckle size first increases and then decreases. Figure 6 shows the surface charge accumulation at the cut-off angle of 180°. The observations are similar to Figure 4 and Figure 5, revealing the presence of a negative charge speckle opposite the metal particle. Moreover, as the charging time increases, the shape of the charge speckle and the position of positive and negative charges remain unchanged. Furthermore, the changes in the maximum positive and negative charge densities and the charge speckle area are consistent with the previous results.

When comparing Figure 4, Figure 5 and Figure 6 for the same duration of applied voltage, the insulator surface charge distribution characteristics at different cut-off phase angles exhibit general similarity, showing no discernible pattern with the change in angle. Numerically, there is no regularity in the difference in maximum positive and negative charge densities among the three cut-off phase angles at the same voltage application time. Furthermore, the interference of minor variables such as temperature, humidity, and measurement error on this difference cannot be ignored. These results indicate that the phase angle of the voltage cut-off moment has a negligible effect on the surface charge accumulation feature when applying AC voltage to the insulator.

### 3.2. Influence of Phase Angle on Charge Quantity

**Figure 7:**
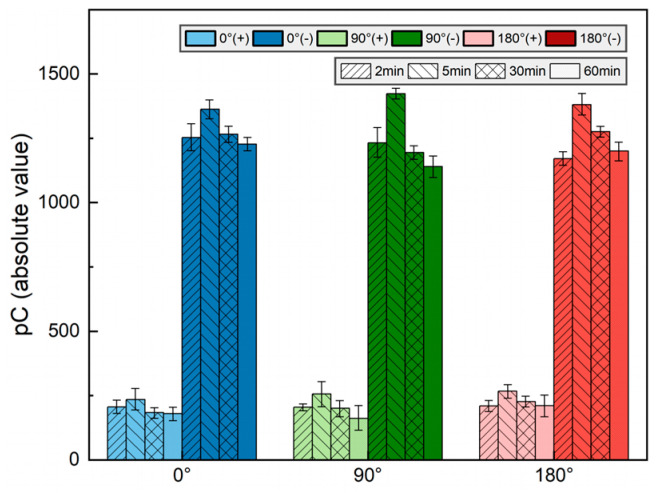
Total measured charges on the surface of insulators.

Figure 7 displays the absolute values of the total positive and negative charges on the insulator surface across different cut-off phase angles and durations of HV application. Each color represents four columns corresponding to the charge quantity of 2, 5, 30, and 60 min of voltage application. It is evident that with the increasing voltage duration, the total charge density exhibits a trend in initially increasing and subsequently decreasing. As can be seen, in the presence of the metal particle at the grounded electrode, the accumulated total negative charge on the insulator surface significantly exceeds the positive charge. Notably, whether concerning the positive or negative charge, the total quantity accumulated on the insulator surface does not markedly alter with the variation in the cut-off phase angle.

## 4. Discussion

### 4.1. Simulation Results

**Figure 8:**
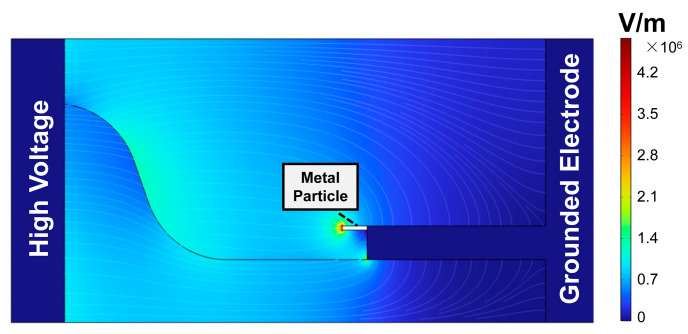
Simulation of the electric field.

A two-dimensional model based on the geometric parameters outlined in Figure 1 was established. The charge accumulation characteristics on the gas–solid interface were analyzed through the COMSOL Multiphysics 6.2 software. Figure 8 presents the simulation results of electric field strength and field lines within the insulator and the mixed gas under the peak of 20 kV AC voltage. It shows that a high electric field strength appears at the tip of the metal particle, reaching a maximum of 4.79 × 10^6^ V/m. The initial discharge field strength of the binary gas mixture is as follows [26]:

(1) $$E_{bd}=p_{a}\cdot E_{a}+p_{b}\cdot E_{b}$$

where *E_bd_* is the initial discharge field strength of the binary gas mixture, *p_a_* and *p_b_* are the partial pressure of components a and b of the gas mixture, and *E_a_* and *E_b_* are the initial discharge field strength of *a* and *b*. The maximum local electric field strength exceeds the calculated value of 4.58 × 10^6^ V/m, which facilitates corona discharge.

The generated positive ions and electrons will move in a directed manner under the effect of the electric field. The non-planar region exhibits a strong normal electric field, mainly directed by the metal particle towards the insulator. However, in the planar region, the tangential electric fields predominate.

According to current research, charges accumulate on the surface of insulators through three transmission modes [27]: conduction on the solid side of the insulator, conduction on the gas side, and conduction on the surface of the insulator. The accumulation process of surface charge can be regarded as the competition between the three conductions.

#### 4.1.1. Solid Side Conduction

The solid side current density of the insulator is the sum of displacement current and convection current:

(2) $$J_{V}=\frac{\partial D}{\partial t}+\gamma_{v}E_{bn}$$

where *J_V_* is the normal current density in the insulator, *D* is electric flux density, *γ_v_* is the volume conductivity of 2 × 10^−16^ S/m of the epoxy insulator, and *E_bn_* represents normal electric field strength on the solid side.

#### 4.1.2. Gas Side Conduction

This model considers the effects of microscopic mechanisms such as generation, diffusion, migration, and the recombination of charged particles in insulating gasses. The current density on the gas side consists of displacement current and conduction current is as follows:

(3) $$J_{G}=\frac{\partial D}{\partial t}+e(\mu^{+}n^{+}+\mu^{-}n^{-})E_{gn}-e\nabla (D^{+}n^{+}-D^{-}n^{-})$$

where *J_G_* is the normal current density in the gas, *E_gn_* is the normal electric field strength in gas, *μ^+^* and *μ*^−^ express the mobility of positive/negative ions, *D^+^* and *D*^−^ represent the ions’ diffusion coefficient, and *n^+^* and *n*^−^ represent the carrier density of the ions.

(4) $$\frac{\partial n^{+}}{\partial t}=n_{ip}-Rn^{+}n^{-}-\nabla \cdot (\mu^{+}n^{+}E)+D^{+}\nabla^{2}n^{+}$$

(5) $$\frac{\partial n^{-}}{\partial t}=n_{ip}-Rn^{+}n^{-}+\nabla \cdot (\mu^{-}n^{-}E)+D^{-}\nabla^{2}n^{-}$$

where *n_ip_* expresses ion pair generation rate and *R* is the recombination coefficient, corresponding to the value of 2.2 × 10^−6^ cm^3^/s.

(6) $$D^{+/-}=\frac{\mu^{+/-}T_{g}K_{b}}{e}$$

where *T_g_* is gas temperature and *K_b_* is the Boltzmann constant.

#### 4.1.3. Surface Conduction

The surface current density of insulators can be expressed by the surface conductivity and tangential electric field:

(7) $$J_{S}=\nabla \cdot (E_{t}\gamma_{s})$$

The temporal change in the surface charge density *σ* can be described as follows:

(8) $$\frac{\partial \sigma }{\partial t}=J_{V}-J_{G}-J_{S}$$

where *J_S_* is surface current density, *E_t_* is the tangential electric field strength, and *γ_s_* is the epoxy insulator surface conductivity of 1.2 × 10^−18^ s.

**Figure 9:**
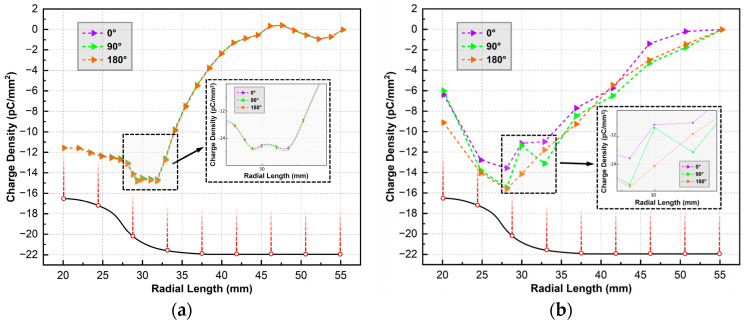
Surface charge density under different turn-off phase angles: (**a**) simulation result; (**b**) experimental result.

In the simulation, the cut-off phase angle is 0° after applying the AC voltage of 50 Hz with an amplitude of 20 kV for 2 min. On this basis, additional 1/4 and 1/2 cycle voltages were applied, corresponding to 90° and 180° cut-off phase angles, respectively. The results are depicted as the surface charge density distribution shown in Figure 9a, with the solid black line outlining the insulator. As can be seen, the most significant charge accumulation occurs near a radial length of 30 mm corresponding to the largest normal electric field strength on the insulator surface. The difference in charge density due to different phase angles in this region can reach 0.14 pC/mm^2^. However, due to the greater variance in the results from the measurement error and small changes in gas composition, temperature, etc., the difference between the measured values is much higher under the same conditions. As can be seen from Figure 9b, the difference in the surface charge density of the insulator due to the phase is up to 2.08 pC/mm^2^. Therefore, the influence of phase angle at the AC voltage cut-off moment on the measurement of insulator surface charge density can be disregarded.

**Figure 10:**
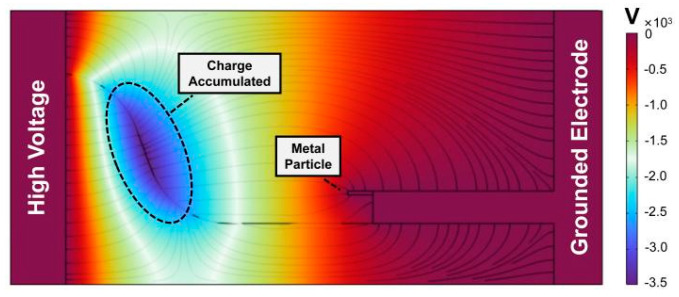
Simulation of the surface charge accumulation.

Figure 10 depicts the potential distribution under the action of the insulator surface charge after the 2 min application of 20 kV AC voltage. It reveals that in the non-planar region opposite the metal particle, the maximum potential can reach −3.49 × 10^3^ V. This phenomenon arises from the substantial accumulation of negative charge in this region.

### 4.2. Analysis of Charge Distribution

**Figure 11:**
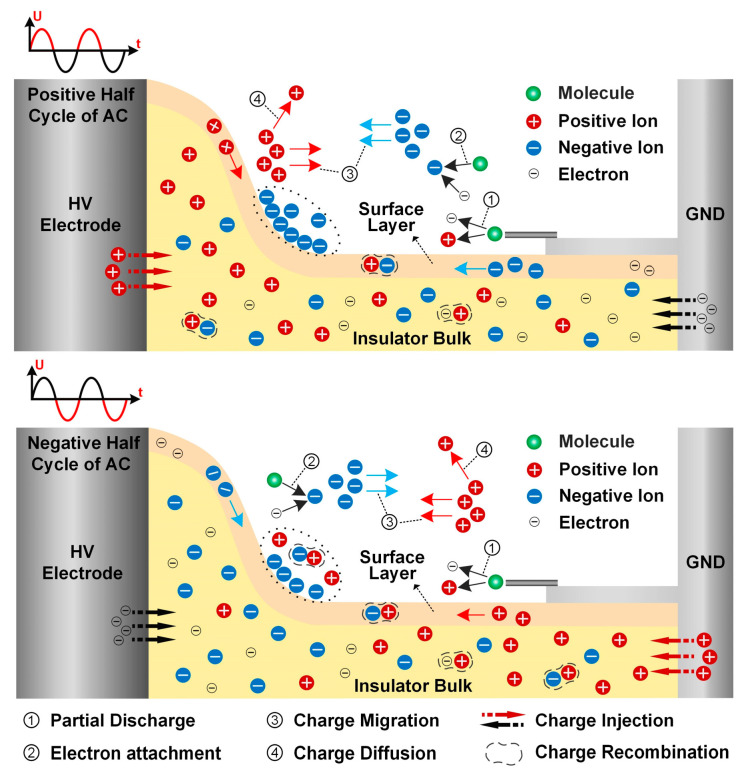
Mechanism diagram of applied AC voltage on surface charge accumulation.

When there is a gas side discharge source, the charge accumulation on the insulator surface is shown in Figure 11. The charge transport of AC voltage at positive and negative polarity is distinguished. Because of the high electric field intensity near the tips of the metal particle, the partial discharge (mark ①) is easily induced, thereby promoting gas ionization to produce a large number of charged particles [28]. These positive ions and electrons migrate toward the insulator surface or grounded electrode under the influence of electric field force. Due to the strong electronegativity of C_4_F_7_N gas, free electrons are easily adsorbed on their outer electron orbitals to form negative ions [29], as depicted by ②. The charged particles migrate in different directions under the influence of the electric field force according to their polarity, as marked by ③. In the process of motion, some ions recombine to form gas molecules, while some ions diffuse along the direction of non-electric field lines (④), and some are attached to the insulator surface and trapped to form surface charges. Furthermore, the polarity of charge injected from electrodes is correlated with the AC voltage.

In the positive half-cycle of the AC voltage, electrons and negative ions on the gas side and positive ions on the solid side migrate toward the insulator surface under the influence of the electric field, especially the non-planar region. With the relatively few charged particles in the insulator bulk and positive ions injected from the HV electrode, the dominant position in surface charge accumulation is occupied by the negative charge on the gas side. Due to the low mass of electrons generated by partial discharge, they swiftly accelerate toward the insulator driven by the electric field. The majority of these electrons will be adsorbed onto C_4_F_7_N molecules, forming negative ions that either deposit on the insulator surface or remain within the gas. During the negative half-cycle, positive ions resulting from partial discharge migrate toward the insulator surface. However, due to their greater mass and slower movement compared to electrons, many positive ions recombine with the negative ions remaining in the gas. Only a few positive ions manage to reach the insulator surface before the voltage reverses again. Consequently, after multiple voltage cycles, a substantial accumulation of negative charge is observed in the non-planar region. Conversely, the plane region exhibits negligible charge accumulation due to the weak normal component of the surface electric field.

As the voltage duration increases, the charge accumulation pattern changes. This phenomenon may be caused by the short-term intense discharge at the initial stage of applied HV; as time progresses, the discharge enters a stable stage [30]. Furthermore, the accumulated surface charge also has approached stability. As a significant number of negative charges accumulate on the non-planar region of the surface, the electric field induced by these charges can inhibit negative particles from approaching the insulator surface. The accumulated charge in this area will decrease in the following cycles. Consequently, as the inhibitory effect weakens, charge accumulation increases again over several cycles. Therefore, in the later stages of applying AC voltage, the surface charge on the insulator remains in dynamic balance, with relatively minor differences in its dynamic changes.

## 5. Conclusions

This work focuses on the effect of cut-off phase angles and AC voltage duration on the surface charge accumulation in the C_4_F_7_N/CO_2_ mix gas with the presence of a metal particle. The findings are summarized as follows:

1. For the insulator, the change in magnitude of the surface charge caused by the phase angle calculated theoretically can reach 0.14 pC/mm^2^. However, the maximum difference in values measured by experiments is 2.08 pC/mm^2^. It can be seen that measurement errors combined with other small changes in variables can lead to much greater measurement differences in experiments. The effect of the cut-off phase angle on the surface charge measurement is only secondary. Therefore, in the subsequent experiments, the control of the phase angle at AC voltage cut-off moment can be disregarded.
2. After applying HVAC of 2, 5, 30, and 60 min, the characteristics of the charge distribution are almost unchanged. The negative charge speckle tends to gather first and then dissipate. In each group of experimental results, the total charge quantity and maximum charge density showed a growth trend in the initial 5 min and then decreased to a dynamic balance subsequently.
3. Under the AC voltage of 20 kV, there are both positive and negative charges accumulated on the insulator. When the metal particle existed in the grounded electrode, the total amount of negative charge on the surface of the insulator will far exceed that of positive charge, and its value can reach 5.54–7.08 times that of the positive charge.

## Data Availability

The original contributions presented in the study are included in the article, further inquiries can be directed to the corresponding author.
